# Postablation Stimulated Thyroglobulin Level is an Important Predictor of Biochemical Complete Remission after Reoperative Cervical Neck Dissection in Persistent/Recurrent Papillary Thyroid Carcinoma

**DOI:** 10.1245/s10434-012-2624-8

**Published:** 2012-09-07

**Authors:** Brian Hung-Hin Lang, Kai Pun Wong, Koon Yat Wan

**Affiliations:** 1 Department of Surgery, The University of Hong Kong, Hong Kong SAR, China; 2 Department of Clinical Oncology, The University of Hong Kong, Hong Kong SAR, China

## Abstract

**Background:**

The efficacy of reoperative cervical neck dissection (RND) in achieving biochemical complete remission (BCR) (or postreoperation stimulated thyroglobulin [sTg] of <0.5 ng/mL) remains unclear in persistent/recurrent papillary thyroid carcinoma (PTC). We hypothesized that lower postablation sTg levels would indicate a higher rate of BCR after RND. Our study examined the association between postablation sTg and BCR after one or more RNDs.

**Methods:**

Of 199 patients who underwent RND, 81 patients were eligible. The postablation sTg levels (≤2 and >2 ng/mL) were correlated with the postreoperation sTg levels after RNDs. Patients’ clinicopathological characteristics, operative findings, and subsequent RNDs were compared between those with BCR after RNDs and those without.

**Results:**

Those with postablation sTg levels of ≤2 ng/mL had significantly higher BCR rate after the first RND (77.8 vs. 5.6 %, *p* < 0.001), overall BCR after one or more RNDs (77.8 vs. 9.3 %, *p* < 0.001), and better 5-year recurrence-free survival after the first RND (80.0 vs. 60.1 %, *p* = 0.049) than those with postablation sTg levels of >2 ng/mL. Overall BCR gradually decreased after each subsequent RND. Postablation sTg significantly correlated with postreoperation sTg (ρ = 0.509, *p* < 0.001). After adjusting for the number of metastatic lymph nodes excised at first RND and presence of extranodal extension, postablation sTg of ≤ 0.2 ng/mL was the only independent factor for BCR after one or more RNDs (odds ratio 37.0, 95 % confidence interval 5.68–250.0, *p* = 0.001).

**Conclusions:**

Only a third of patients who underwent one or more RNDs for persistent/recurrent PTC had BCR afterward. Postablation sTg level was an independent factor for BCR. Completeness of the initial operation is important for the subsequent success of RND.

Papillary thyroid carcinoma (PTC) is the most common type of differentiated thyroid carcinoma, and its age-adjusted incidence has doubled in the last 25 years.^1^ Despite its relatively good prognosis, with a 10-year cancer-specific survival above 90 %, locoregional recurrence is common.^2^ Local recurrences are found in 5–20 % of patients with PTC, of which two-thirds are localized in the cervical lymph nodes.^2^ For patients treated with total thyroidectomy and radioiodine (RAI) ablation in whom all normal thyroid tissue has been ablated, disease monitoring or surveillance for persistent/recurrent disease relies on measurement of thyroglobulin (Tg) and on high-resolution neck ultrasound (USG).^3^ Both basal Tg and postablation stimulated Tg (sTg) by T4 withdrawal or recombinant human thyroid-stimulating hormone injection are accurate predictors for future persistent/recurrent disease.^4^^–^ 7 In the absence of distant metastases, a single postablation sTg value of >2 ng/mL indicates a high possibility of residual disease.^3^^,^ 6 Furthermore, the use of high-resolution USG has increased the identification of small-volume/nonpalpable neck lymph node metastases. However, the benefit of surgically removing these asymptomatic small volumes of metastatic lymph nodes remains unclear.^3^ The American Thyroid Association recommends surgical removal of clinically significant metastatic lymph nodes to prevent future locoregional complications.^3^^,^ 8 Therefore, the long-term efficacy of reoperative cervical neck dissection (RND) in terms of local control and biochemical remission (defined by postreoperation sTg) remains controversial. A few studies have reported the efficacy of RND by evaluating the postreoperation sTg.^8^^–^ 11 A postreoperation sTg level of <0.5 ng/mL or biochemical complete remission (BCR) is an accurate surrogate marker for long-term outcomes after RND.^9^ However, the rate of attaining BCR after first or multiple RNDs varied between studies and factors for BCR after RND remained undefined.^8^^–^ 11

Because most persistent/recurrent disease probably represents residual disease, we hypothesized that the postablation sTg value might predict BCR after one or more RNDs.^6^^,^ 7 ^,^ 9 Our study aimed to evaluate the efficacy of RND in achieving BCR and to determine factors for BCR after one or more RNDs.

## PATIENTS AND METHODS

A retrospective review was performed on all patients who underwent RND for locally persistent/recurrent PTC from 1996 to 2008. Before RND, all already had complete removal of the thyroid gland either at our institution or elsewhere. Some patients also had concomitant neck dissection involving the central (level VI) and/or lateral compartment (levels II–V). A standard dose of 3 GBq RAI ablation was given to all patients 2–3 months after the initial operation. A sTg level was then checked 6–9 months after the ablation (i.e., the postablation sTg level). After that, patients were placed on a surveillance protocol with Tg monitoring and USG.^12^^,^ 13 Persistent/recurrent PTC was suspected on the basis of factors like rising trend of unstimulated Tg, suspicious sonographic lymph node features such as hyperechoic punctuations, cystic appearance, hypervascularization, and round-shaped node without fatty hilum and/or positive fine-needle aspiration cytology findings.^4^^,^ 14 Once persistent/recurrent PTC was diagnosed, further characterization by computed tomography, fluorodeoxyglucose–positron emission tomography/computed tomography, and/or whole body scan was performed. For those with imageable locoregional persistent/recurrent disease, RND was generally preferred over observation or RAI alone, regardless of the volume of detectable disease. We generally did not proceed to reoperation unless there was a positive localization study. Two months after RND, a further dose of RAI ranging between 3 and 5.5 GBq was given as an adjuvant treatment, depending on the extent of disease. A postreoperation sTg level was then taken 6–9 months after RAI therapy. For patients requiring two or more RNDs, a postreoperation sTg was taken each time. BCR was defined as postreoperation sTg of <0.5 ng/mL. When calculating the overall BCR, only the last reoperation sTg value was taken when the patient had multiple RNDs.

In order to evaluate the efficacy of RND on postreoperation sTg, patients with proven or suspected distant metastases as evident by imaging or with known residual gross disease after initial operation were excluded. Anti-Tg antibodies were routinely checked for all patients. Patients with anti-Tg antibodies or with a missing pair of postablation and postreoperation sTg levels were also excluded. All data relating to the initial operation and subsequent RNDs were collected prospectively, and follow-up data were regularly updated in a computerized database. The present study protocol was approved by the local institutional review board.

### Surgical Resection for Persistent/Recurrent PTC

Patients with persistent/recurrent PTC underwent either formal central (VI), lateral (levels II–V), or both neck dissections for lymph node recurrence on the basis of the findings of the preoperative imaging studies. The central compartment was not routinely explored at the time of reoperation unless there was suspected or proven disease in the area. An en-bloc or compartmental neck dissection was preferred unless certain compartments had been previously dissected, or a focused neck dissection or completion compartmental neck dissection was performed. Before surgery, the exact location of metastatic lymph nodes was marked on the neck via USG.

### Tg Measurement

All Tg levels were measured in the same laboratory using the same immunometric assay. The assay used was the Immulite 2000 (Diagnostic Products Corporation, Roche, Los Angeles, CA). This was calibrated against the CRM-457 standard. Normal reference range was <0.5–55 μg/L, and sensitivity was <0.2 μg/L.

### Statistical Analysis

Statistical analysis was performed by χ^2^ or Fisher’s exact tests to compare categorical variables, and the Mann–Whitney *U*-test was used to compare continuous variables between groups. Variables significant in the univariate analysis were entered into the multivariate analysis. To improve clinical utility, before entering into the multivariate analysis, potentially significant continuous variables were converted into categorical variables using their own median as the cutoff. Binary logistic regression analysis with a variable entrance criterion of 0.05 or less was conducted to identify factors associated with BCR after one or more RND. Recurrence-free survival was calculated by the Kaplan–Meier method, and survival rates were compared by the log-rank test. The Spearman rank correlation test was used to correlate postablation sTg with postreoperation sTg. All statistical analyses were performed by SPSS software, version 18.0 (SPSS, Chicago, IL).

## Results

There were 243 consecutive RNDs performed on 199 patients over the study period. Sixty-four patients (32.1 %) were excluded because of distant metastases (*n* = 33), gross residual tumor left behind (*n* = 19), or obvious bulky residual nodal disease detected at initial scan (*n* = 12). Forty patients (20.1 %) with anti-thyroglobulin antibodies and another 14 patients (7.0 %) with a missing pair of postablation and postreoperation sTg levels were also excluded. Therefore, 81 patients (40.7 %) with 106 RNDs were eligible for analysis. Table 1 shows the patients’ baseline characteristics. All the initial selective neck dissections were performed with therapeutic intent (i.e., resection of clinically evident lymph node metastases, or cN1). None had undergone prophylactic central neck dissection. After surgery, all patients received RAI ablation, and of these, eight patients received one further dose of RAI because of elevated postablation sTg without imageable residual disease. The median cumulative dose of RAI was three (range 3–7.5) GBq, and the final median postablation sTg level was 12.3 (<0.2–126) ng/mL. Twenty-seven patients (33.3 %) had postablation sTg values of ≤2 ng/mL. The median time interval from initial surgery to first RND was 33.6 (range 13.1–286.9) months, and the median follow-up period from initial surgery to time of analysis was 89.5 (range 28.1–375.7) months. The most common method of diagnosing persistent/residual disease was by USG without fine-needle aspiration cytology (50.6 %). All patients had proven PTC on histology. Of the patients requiring RND in the lateral compartment alone, 34 (59.6 %) of 57 already had initial formal lateral neck dissection, and of those requiring RND in the central compartment alone, 1 (12.5 %) of eight had initial formal central neck dissection.

**TABLE 1:** Baseline characteristics of 81 patients with RND

Characteristic	Value
Age at initial operation (year)	42.6 (11.8–85.3)
Age at first RND (year)	45.2 (13.1–87.4)
Interval between initial operation to first RND (month)	33.6 (13.1–286.9)
Gender	
Male	26 (32.1 %)
Female	55 (67.9 %)
Type of initial operation	
Total thyroidectomy	73 (90.1 %)
Completion total thyroidectomy	4 (4.9 %)
Lobectomy followed by completion total thyroidectomy	4 (4.9 %)
Type of initial concomitant neck dissection	
Central compartment	10 (12.3 %)
Lateral compartment	24 (29.6 %)
Central and lateral compartments	24 (29.6 %)
Tumor stage of primary PTC by TNM stage	
I	47 (58.0 %)
II	14 (17.3 %)
III	18 (22.2 %)
IV	2 (2.5 %)
Tumor characteristics	
Size (cm)	2.0 (0.7–9.0)
Extrathyroidal extension	29 (35.8 %)
Tumor multifocality	45 (55.6 %)
Capsular invasion	39 (48.1 %)
Lymph node metastases (pN1)	65 (80.2 %)
Postablation stimulated thyroglobulin after initial operation (ng/mL)	12.3 (<0.2–126)
Method of diagnosing persistent/recurrent PTC before first RND	
Ultrasound without FNAC	41 (50.6 %)
Ultrasound guided FNAC	20 (24.7 %)
CT/MRI or FDG-PET/CT	20 (24.7 %)
Type of first RND	
Focused or completion dissection	48 (59.3 %)
Formal neck dissection	33 (40.7 %)
Extent of first RND	
Central compartment only	8 (9.9 %)
Lateral compartment only	57 (70.4 %)
Central and lateral compartments	16 (19.8 %)
No. of RNDs performed	
1	65 (80.2 %)
2	9 (11.1 %)
3	5 (6.2 %)
4	2 (2.5 %)
5	0 (0.0 %)

Data are presented as median (range) or *n* (%)

*RND* reoperative cervical neck dissection, *PTC* papillary thyroid carcinoma, *TNM* tumor, node, metastasis staging system, *FNAC* fine-needle aspiration cytology, *CT* computed tomography, *MRI* magnetic resonance imaging, *FDG*–*PET* fluorodeoxyglucose–positron emission tomography

**Fig. 1:**
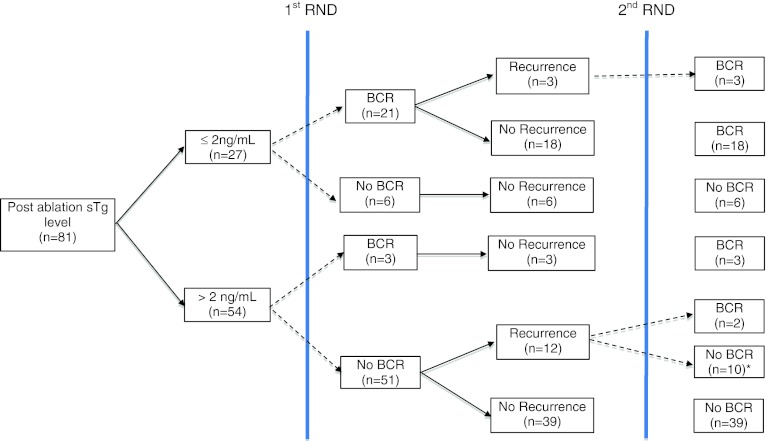
Flowchart of the 81 patients who required one or more reoperative cervical neck dissections according to postablation stimulated thyroglobulin values of ≤2 and >2 ng/mL. * 7 out of 10 patients required ≥3 reoperations. Abbreviations: *BCR* biochemical complete remission, *sTg* stimulated thyroglobulin, *RND* reoperative cervical neck dissection

**Fig. 2:**
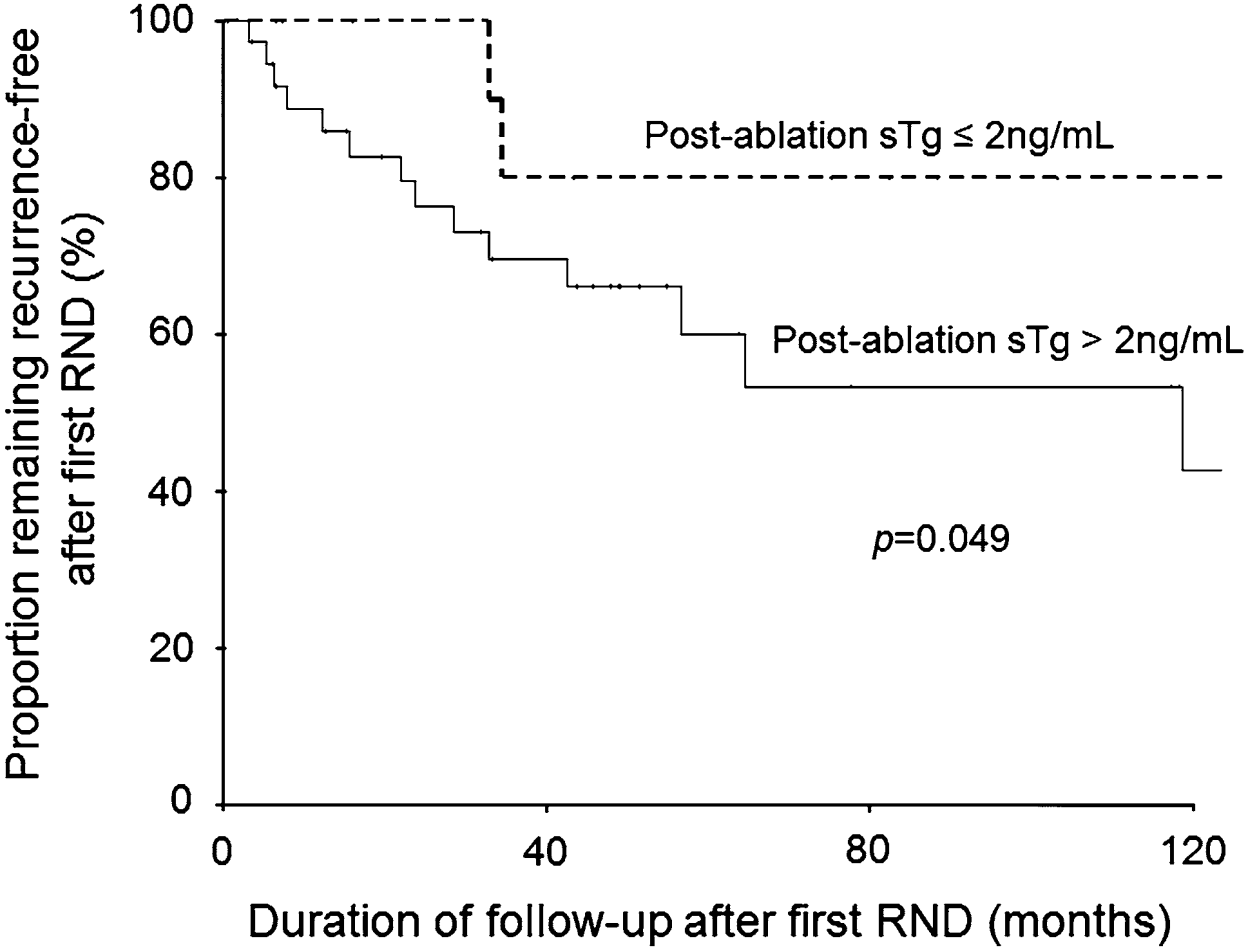
Recurrence-free survival curves after first reoperative cervical neck dissection between postablation stimulated thyroglobulin values of ≤2 and > 2 ng/mL

Figure 1 shows the breakdown of the 81 patients according to the level of postablation sTg levels of ≤2 and >2 ng/mL. The group with postablation sTg of ≤2 ng/mL had significantly higher BCR rates after the first RND (77.8 vs. 5.6 %, *p* < 0.001) and overall BCR after one or more RNDs (77.8 vs. 9.3 %, *p* < 0.001) than the group with postablation sTg of >2 ng/mL. The recurrence-free survival from initial operation also tended to be longer than the group with postablation sTg of >2 ng/mL (41.1 vs. 27.8 months, *p* = 0.071). The 5-year recurrence-free survival after first RND also was significantly better than the group with postablation sTg of >2 ng/mL (80.0 vs. 60.1 %, *p* = 0.049). Figure 2 shows the recurrence-free survival curves after first RND between postablation ≤2 and >2 ng/mL.

The overall rate of BCR after first RND was 24 (29.6 %) of 81; the rate of BCR after second RND was 5 (33.3 %) of 15; the rate of BCR after third RND was 0 (0 %) of seven. The overall rate of BCR after RNDs was 26 (32.1 %) of 81. At the time of analysis, these 26 patients did not have detectable persistent/recurrent PTC.

Table 2 shows a comparison of primary tumor characteristics, postablation sTg, and findings at the first RND between those who experienced BCR after one or more RNDs (BCR-positive group) and those who did not achieve BCR after one or more RNDs (BCR-negative group). The age at initial operation and the age at first RND in the BCR-negative group tended to be older than in the BCR-positive group (*p* = 0.186 and *p* = 0.077, respectively). Compared to the BCR-negative group, the BCR-positive group was significantly more likely to have postablation sTg of ≤2 ng/mL (80.8 vs. 10.9 %, *p* < 0.001), a higher number of metastatic lymph nodes excised at first RND (6 vs. 3, *p* = 0.039), and absent extranodal extension (3.8 vs. 52.7 %, *p* < 0.001). The BCR-positive group had significantly lower median postablation sTg than the BCR-negative group (0.5 vs. 17.0 ng/mL, *p* < 0.001). Postablation sTg significantly correlated with postreoperation sTg (ρ = 0.509, *p* < 0.001).

**TABLE 2:** Comparison of characteristics after initial operation and findings at first reoperation

Characteristic	BCR-positive group	BCR-negative group	*p*
	(*n* = 26)	(*n* = 55)	
Age at initial operation (year)	30.4 (18.3–59.9)	43.1 (11.8–85.3)	0.186
Age at first RND (year)	34.6 (19.9–60.2)	49.2 (13.1–86.5)	0.077
Gender (M:F)	7:19	19:36	0.604
Primary tumor stage by TNM stage			0.781
I	16 (61.5)	31 (56.4)	
II	5 (19.2)	9 (16.4)	
III	5 (19.2)	13 (23.6)	
IV	0 (0.0)	2 (3.6)	
Primary tumor characteristics			
Tumor size (cm)	2.3 (0.8–5.0)	2.0 (0.7–9.0)	0.442
Extrathyroidal extension	12 (46.2)	27 (49.1)	0.767
Tumor multifocality	14 (53.8)	31 (56.4)	0.923
Capsular invasion	9 (35.6)	30 (54.5)	0.148
LN metastasis (pN1)	19 (73.1)	46 (83.6)	0.345
Tg level			<0.001*^,b^
After ablation (ng/mL)	0.5 (<0.2–9.7)	7 (1.6–126.0)	
≤2 ng/mL	21 (80.8)	6 (10.9)	
>2 ng/mL	5 (19.2)	49 (89.1)	
Type of first RND			0.114
Focused/completion	12 (46.2)	36 (65.5)	
Formal dissection	14 (53.8)	19 (34.5)	
Extent of first RND			0.144
Central compartment only	4 (15.4)	4 (7.3)	
Lateral compartment only	20 (76.9)	37 (67.3)	
Central and lateral compartments	2 (7.7)	14 (25.5)	
No. of LNs excised at first RND	13 (3–42)	11 (5–50)	0.767
No. of positive LNs excised at first RND	6 (1–12)	3 (1–17)	0.039*^,b^
LN ratio (%)^a^	43.3 (33.3–53.3)	32.5 (13.3–60.0)	0.326
Presence of extranodal extension	1 (3.8)	29 (52.7)	<0.001*^,c^
Postreoperation sTg level after first RND (ng/mL)	0.2 (<0.2–3.0)	17 (0.9–134.1)	<0.001*^,b^

Data are presented as median (range) or *n* (%). Name of test has been added for each significant variable

*BCR*-*positive* biochemical complete remission, *BCR*-*negative* no biochemical complete remission, *RND* reoperative cervical neck dissection, *LN* lymph node, *sTg* stimulated thyroglobulin level

^a^LN ratio = (Number of positive lymph nodes excised/number of lymph nodes excised) × 100

^b^Mann-Whitney U test

^c^Fisher’s Exact test

* Statistically significant at *p* < 0.05

Table 3 shows a multivariable analysis for BCR after RNDs. After adjusting for the number of metastatic lymph nodes excised at first RND (≥4 or <4) and extranodal extension, postablation sTg of ≤0.2 ng/mL was the only independent factor or predictor of BCR after RNDs (odds ratio 37.0, 95 % confidence interval 5.68–250.0, *p* = 0.001). Because age at first RND almost reached significant in the univariate analysis, it was entered as a continuous variable in the multivariate analysis. However, postablation sTg of ≤0.2 ng/mL remained the only independent factor or predictor of BCR after RNDs (odds ratio 37.0, 95 % confidence interval 5.21–250.0, *p* < 0.001).

**TABLE 3:** Multivariable analysis for achieving overall biochemical complete remission after one or more RNDs

Covariate	Odds ratio	95 % Confidence interval	*p*
Postablation stimulated thyroglobulin^a^			0.001
>2 ng/mL	1		
≤2 ng/mL	37.0	5.68–250.0	
No. of metastatic lymph nodes excised at first RND			0.227
≥4	1		
<4	3.24	0.48–21.92	
Extranodal extension at first RND			0.998
Absent	1		
Present	1.9 × 10^9^	0.00001–1 × 10^12^	

*RND* reoperative cervical neck dissection for nodal recurrence

^a^Postablation stimulated thyroglobulin remained significant (*p* < 0.05) when age at first RND was entered into the multivariate analysis

## Discussion

Although RND is an effective and safe treatment option for persistent/recurrent PTC, the efficacy of RND in achieving long-term recurrence-free survival remains uncertain.^8^^,^ 15 On the basis of the current recommendation, the primary aim of RND in persistent/recurrent PTC is to prevent future locoregional complications because only 30–50 % of patients would remain free of disease in the short term after RND.^3^^,^ 6 Similar to other studies, our study used BCR as a surrogate marker for long-term outcomes after RND.^8^^–^ 11 Our overall rate of BCR after one or more RND was 32.1 %, which appeared to be within the range of 21–51 % reported in other studies. However, unlike other studies, our study showed that after adjusting for the number of metastatic lymph nodes at first RND and presence of extranodal extension, postablation sTg level after initial operation was an independent factor for BCR after one or more RNDs. Because postablation sTg of >2 ng/mL usually indicates residual disease after initial treatment, 2 ng/mL was chosen as the cutoff. We found that those with postablation sTg of ≤2 ng/mL had a BCR rate of 77.8 %, whereas those with postablation sTg of >2 ng/mL had a BCR rate of only 5.6 % after first RND. This agreed with the findings of a recent study that found that a lower prereoperation sTg level was significantly associated with a higher BCR rate.^9^

Although our study did not examine the association between postablation sTg and prereoperation sTg or between prereoperation sTg and BCR rate, we would expect there would be a significant association between postablation and prereoperation sTg because the majority of persistent/recurrent disease probably represents subclinical residual disease from initial treatment.^6^^,^ 7 ^,^ 16 This might also explain the significant association between postablation sTg and postreoperation sTg. The group with postablation sTg of ≤2 ng/mL probably started with a significantly lower volume of residual disease than the group with sTg >2 ng/mL, and when the former group developed detectable recurrence and required RND, the volume of disease remained relatively small and so could be more completely excised by surgery. In contrast, by the time the group with postablation sTg of >2 ng/mL required RND, the extent of persistent/recurrent PTC might have progressed beyond local repeat resection. This might explain why the group with sTg of >2 ng/mL had a significantly lower BCR rate and a significantly better 5-year recurrence-free survival after first RND in the group with postablation sTg of ≤2 ng/mL than the group with sTg of >2 ng/mL (*p* = 0.049).

Similar to a previous study, we noticed the rate of achieving BCR gradually decreased with each subsequent RND.^11^ None of the seven patients who had three or more RNDs experienced BCR, although they remained free of disease at the time of analysis. Our data indicated that the efficacy of the second reintervention seemed to depend on the level of postablation sTg. If the postablation sTg was ≤2 ng/mL, the efficacy of the second RND for BCR remained 100 % successful (i.e., 3 of 3), whereas if the postablation sTg was >2 ng/mL, the efficacy of the second RND was only 2 (16.7 %) of 12. There are several explanations for this. First, locoregional persistent/recurrent disease may progress over time, and it may become impossible to be completely removed by local resection. Second, after each reoperation, more scarring is generated in the operating field, which makes each subsequent RND more limited and difficult. Although surgical complications were not specifically evaluated, we think that the decision for multiple RNDs should be balanced between increasing surgical risks and decreasing chance of complete resection or BCR. We were not able to identify any additional preoperative clinical factors for BCR. The two clinical factors associated with BCR—number of positive lymph nodes excised and extranodal extension—were postoperative factors only.

Despite these findings, our study had several shortcomings. First, the number of patients was relatively small, which limited the power of the study. Also, because of the exclusion criteria, over half of the consecutive patients had to be excluded, so our cohort represented a highly selected patient group. Because none of the patients underwent concomitant prophylactic central neck dissection at the initial operation, it remains unknown whether this dissection might have affected the BCR rate after RND. Preoperative USG is known to have a low sensitivity in detecting central compartment metastases.^12^^,^ 17 Also, because we did not routinely explore the central compartment at the time of RND unless there was suspected disease, it was unknown whether there was still subclinical residual disease in the central compartment and whether clearing this compartment might further improve the BCR rate. Because the data for prereoperative sTg levels were not available, our findings and postulations await confirmation by future studies.

In conclusion, only a third of patients who underwent RND for persistent/recurrent PTC experienced BCR afterward. Postablation sTg level significantly correlated with postreoperation sTg level. After adjusting for the number of metastatic lymph nodes at first RND and the presence of extranodal extension, postablation sTg level was an independent factor for BCR after one or more RNDs. Our findings highlight the importance of the completeness of the first operation on the subsequent success of RNDs. Concomitant prophylactic central neck dissection may play a critical role in achieving better completeness in the first operation.
